# Finite element biomechanics of novel intramedullary nails with varying diameters for intertrochanteric femoral fractures with lateral wall injury

**DOI:** 10.3389/fbioe.2026.1758146

**Published:** 2026-02-24

**Authors:** En Wu, Zhongjian Tang, Liucheng Wang, Haitao Liu, Zhiwei Peng, Yonghui Liang

**Affiliations:** 1 Department of Emergency Trauma Center, Aerospace Center Hospital, Beijing, China; 4 Department of Orthopaedics, The Second Affiliated Hospital of XuZhou Medical University, Xuzhou, China; 2 Graduate School of Hebei Medical University, Shijiazhuang, China; 3 Department of Traumatology and Orthopedics, Peking University People’s Hospital, Beijing, China

**Keywords:** finite element analysis, intertrochanteric femoral fractures, lateral wall injury, proximal femoral bionic nail, proximal femoral total bionic nail

## Abstract

**Objective:**

This study aimed to compare the fixation performance differences between the traditional proximal femoral nail antirotation (PFNA), “II” proximal femoral bionic nail (PFBN), and proximal femoral total bionic nail (PFTBN) in the treatment of intertrochanteric femoral fractures complicated by lateral wall injury using finite element analysis. Additionally, it intended to explore the biomechanical effects of reamed versus unreamed techniques on these novel intramedullary nail devices.

**Methods:**

Validated FEA models of intertrochanteric fractures (lateral wall injury) were constructed with 9 mm (unreamed) and 11 mm (reamed) medullary canals, followed by implantation of PFNA, “II” PFBN, or PFTBN. Under a vertical load of 2100 N, Von Mises Stress (VMS) and displacement of the femur and implants were quantified.

**Results:**

Under a vertical load of 2100 N, in the unreamed condition, the PFTBN exhibited the lowest peak stress and displacement among the three devices, followed by the “II” PFBN, while the traditional PFNA showed the poorest performance. After reaming, all three implants demonstrated increased peak stress and slightly elevated peak displacement; however, PFTBN remained the most stable. Notably, reaming significantly reduced the overall peak stress of the femur. Collectively, PFTBN more effectively reduced the stress and displacement of both the femur and the implant under both reamed and unreamed conditions, with “II” PFBN showing intermediate efficacy. Both novel devices provided superior internal fixation stability compared to PFNA, which may contribute to a reduced risk of postoperative complications.

**Conclusion:**

PFTBN outperforms “II” PFBN and PFNA in load/shear resistance for intertrochanteric fractures with lateral wall injury, regardless of reaming. “II” PFBN also shows superior stability to PFNA. Reaming increases nail-bone contact, mitigating femoral stress concentration and refracture risk. Both PFTBN and “II” PFBN are reliable fixation options with promising clinical utility.

## 1 Introduction

With the intensification of global population aging, the incidence of hip fractures has risen sharply, with approximately 1.6 million cases reported worldwide each year and a projected increase to 4.5–6.3 million by 2050 (Huang et al., 2022; Kokoroghiannis et al., 2012). Hip fractures are the most harmful fracture type in the elderly due to their high mortality rate. In particular, sliding hip screws yield suboptimal fixation outcomes for intertrochanteric femoral fractures complicated by lateral wall injury (Hsu et al., 2013; Fan et al., 2021; Fan et al., 2022). Studies have confirmed that the femoral lateral wall—defined anatomically as the lateral cortical bone of the proximal femur extending to the lateral crest, connecting the greater trochanter and continuing to the midplane of the lesser trochanter (Im et al., 2005; Palm et al., 2007)—is crucial for fracture stability. Approximately 33% of intertrochanteric fractures are accompanied by lateral wall defect (Li et al., 2019), and neglect of such injury may lead to severe complications. Therefore, optimizing the biomechanical performance of internal fixation devices is of great importance.

Currently, the proximal femoral nail anti-rotation (PFNA) is widely used in clinical practice. Featuring a single-head nail design, it has suboptimal antirotational performance and is associated with issues such as insufficient stability of the proximal fracture fragment, as well as loosening and breakage of the screw (Queally et al., 2014; Grønhaug et al., 2022; Brox et al., 2015). To address these defects, researchers have developed the second-generation proximal femoral blade nail (II-PFBN) and the proximal femoral triflange blade nail (PFTBN). Preliminary studies suggest that these devices may have potential advantages in antirotation and lateral wall support, yet comparative biomechanical studies on their application for intertrochanteric fractures with lateral wall injury remain scarce.

In addition, the choice between reamed and unreamed intramedullary nailing remains controversial. Reaming can increase the nail-bone contact area and promote fracture healing (Penzkofer et al., 2009; Rosa et al., 2019), but it is prone to damaging the medullary blood supply and inducing osteonecrosis or fat embolism syndrome (Pairon et al., 2015; Xu et al., 2019). At present, relevant research on biomechanical analyses of novel intramedullary nails for the aforementioned fractures, as well as mechanical comparisons between reamed and unreamed techniques, is relatively limited.

In this study, finite element analysis (FEA) was adopted, with PFNA as the control, to compare the stress distribution and stability of II-PFBN and PFTBN in the fixation of intertrochanteric femoral fractures with lateral wall injury under both reamed and unreamed conditions. The study aimed to clarify the biomechanical differences among the aforementioned devices, fill the research gap in dedicated biomechanical studies of novel intramedullary nails for fractures with lateral wall injury, and provide a theoretical basis for the clinical selection of internal fixation devices and nailing techniques.

## 2 Materials and methods

### 2.1 Construction of 3D models of the femur and intramedullary nail devices

A healthy male volunteer (50 years old, body weight 72 kg) was recruited, and no hip joint-related diseases were confirmed by X-ray examination prior to data collection. Computed tomography (CT) scanning of the left femur was performed with the following parameters: voltage 70–140 kV and current 30–800 mA. The scanning data were stored in Digital Imaging and Communications in Medicine (DICOM) format for subsequent use. The DICOM-format CT data were imported into Mimics 21.0 software (Materialise, Leuven, Belgium) for bone tissue segmentation based on Hounsfield Units (HU). Referring to the research results of Abdul-Wahab et al. (2020), a threshold of 700 HU was set to distinguish cortical bone from cancellous bone. A preliminary three-dimensional (3D) femoral model was constructed and exported as a Stereolithography (STL) file.

The STL file was imported into Geomagic software (3D Systems, Rock Hill, SC, USA) to repair surface defects and achieve model closure through mesh derivation. Further optimization of the geometric morphology of the femoral complex surface was performed using polygon fitting, Non-Uniform Rational B-Spline (NURBS) surface reconstruction, and mesh relaxation techniques. The standardized surface model was finally exported in Initial Graphics Exchange Specification (IGES) format. The IGES-format model was imported into ProEngineer 5.0 software (PTC, Boston, MA, USA) for sequential processes including model smoothing, meshing, noise removal, and surface adaptability optimization to construct a high-precision 3D solid femoral model. Subsequently, the solid model was imported into SolidWorks 2017 software (Dassault Systèmes, Vélizy-Villacoublay, France), and independent 3D models of cortical bone and cancellous bone were generated via Boolean operations.

**FIGURE 1:**
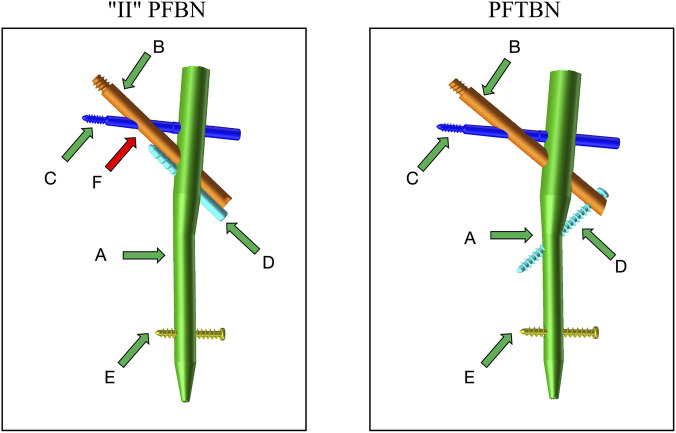
In the “II” PFBN, component A is the main nail, B is the compression screw, C is the tension screw, which is perpendicular to the main nail, D is the pull-out screw, E is the distal locking screw,and F is the fulcrum for both the pressure screw and the pull-out screw. Compared to the first-generation PFBN, the advancement of this fulcrum forward optimizes the mechanical performance. In the PFTBN, component A is the main nail, B is the compression screw, C is the tension screw, which is perpendicular to the main nail, D is the lateral wall screw, placed from the lateral side of the greater trochanter, and E is the distal locking screw.

**FIGURE 2:**
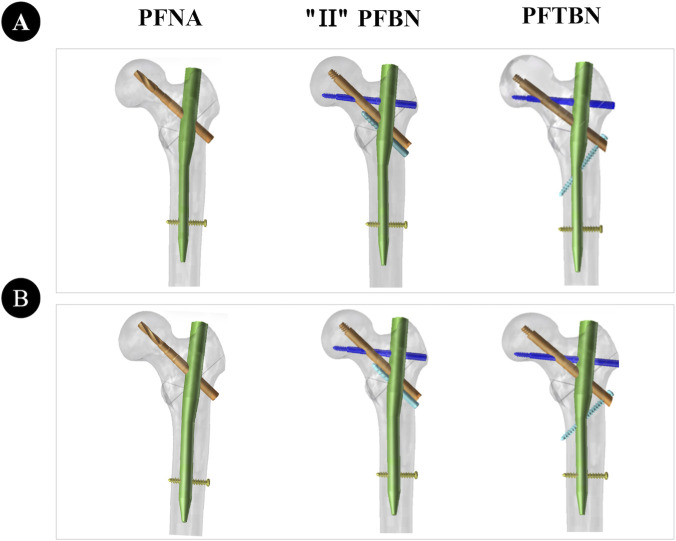
Figure shows the three intramedullary nail device-fracture models. **(A)** presents the intramedullary nail device with a 9 mm main nail under the unreamed condition, while **(B)** displays the intramedullary nail device with an 11 mm main nail under the reamed condition.

Referring to relevant literature (Li et al., 2023), a standardized model of intertrochanteric femoral fracture with lateral wall injury was established in SolidWorks 2017 software: the thickness of the lateral wall was set to 10 mm, model segmentation was completed along a preset fracture line, and the lesser trochanter was resected to simulate typical clinical injury characteristics. Based on clinical fixation specifications and engineering geometric parameters, three types of intramedullary nail internal fixation device models were designed and generated using SolidWorks 2017 software, namely, PFNA, “II” PFBN, and PFTBN (Figure 1). After verifying format compatibility, the three intramedullary nail models were assembled with the fracture model respectively to form a complete fracture-intramedullary nail finite element analysis model (Figure 2).

### 2.2 Material properties

The femur and intramedullary nail devices were assumed to be homogeneous, isotropic, and linearly elastic materials. Titanium alloy was specified as the material for the intramedullary nail devices. Based on previous literature (Chen et al., 2023; Baca et al., 2008; Papini et al., 2007), the Young’s modulus of cortical bone and cancellous bone was set to 16.8 GPa and 0.58 GPa, respectively, with a Poisson’s ratio of 0.3 for both. The Young’s modulus and Poisson’s ratio of the intramedullary nails were 110 GPa and 0.31, respectively. The material parameters of each component are presented in table (Table 1).

**TABLE 1:** Material parameters.

Materials	Elastic modulus (GPa)	Poisson’s ratio
Cortical bone	16.8	0.3
Cancellous bone	0.58	0.3
Head of femur	0.9	0.29
Collum femoris	0.62	0.29
Internal fixation device	110	0.31

### 2.3 Boundary conditions and loading setup

In this study, the bone-screw and bone-bone interfaces were defined as surface contact with a friction contact type. Specifically, the friction coefficient between bone and bone was set to 0.46, between bone and screws to 0.42, and between screws to 0.2, which is consistent with common biomechanical simulation settings for orthopedic implants. For boundary conditions, all degrees of freedom at the distal end of the femoral model were fully constrained, and the contact area between the femoral head and the pelvis was coupled to a single central point for external load application. The loading condition was set to 2100 N, with the direction simulating the vertical downward force during normal standing, which approximates three times the body weight and is a widely used physiological load in femoral finite element studies.

### 2.4 Model validation strategy

To ensure model accuracy, a complete femoral model was first constructed and assigned corresponding material properties according to the methods described in previous literature (Haidukewych, 2009; Mohebbi et al., 2025). Subsequently, all degrees of freedom at the distal end of the femoral model were fully constrained, and a vertical load of 2100 N was applied to the femoral head. In-depth model analysis was performed using Ansys 19.0 software (ANSYS, Inc., Canonsburg, PA, USA), and the obtained results were carefully compared with those reported in the literature (Haidukewych, 2009; Mohebbi et al., 2025) to verify model validity. The stress distribution and displacement characteristics of the validated model were consistent with physiological biomechanical principles, confirming its reliability for subsequent analysis.

### 2.5 Main evaluation parameters

Detailed biomechanical analysis of the femur and the three different intramedullary nail devices was performed using ANSYS Workbench 2020R2 software (ANSYS, Inc., Canonsburg, PA, USA). The output parameters mainly included the Von Mises Stress distribution map of the intramedullary nail devices, displacement distribution map, and overall stress distribution data of the femur. These parameters provided comprehensive and in-depth insights into the mechanical characteristics of the models, enabling quantitative comparison of fixation stability and stress transfer efficiency among different devices under reamed and unreamed conditions.

## 3 Results

### 3.1 Von Mises Stress and displacement distributions of three intramedullary nail devices and the femur under unreamed condition

#### 3.1.1 Von Mises Stress distribution of the three intramedullary nail devices

**FIGURE 3:**
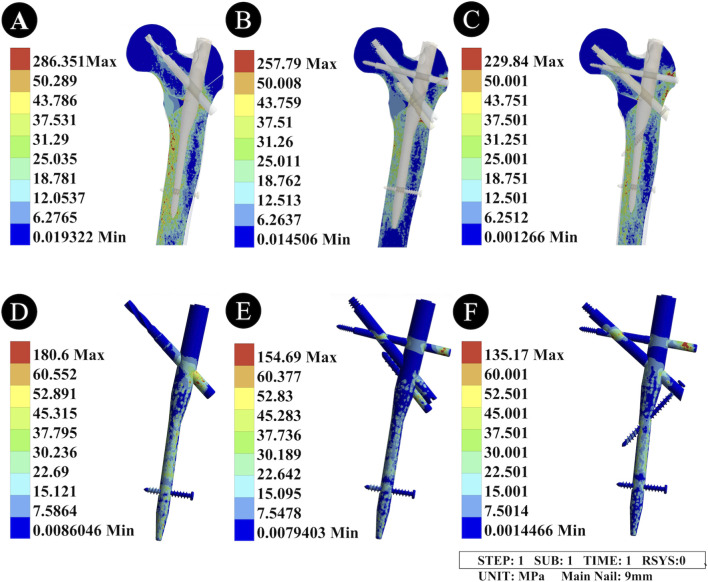
Von Mises Stress distribution diagrams of the femur and intramedullary nail devices under the unreamed condition. **(A,D)** Stress distribution diagrams of the PFNA device; **(B,E)** Stress distribution diagrams of the “II” PFBN device; **(C,F)** Stress distribution diagrams of the PFTBN device. A-C represent the Von Mises stress (VMS) distribution of the three intramedullary nail devices, with stress concentration at the junctions of the tension screw and lateral wall, and compression screw and tension screw; the peak VMS values are 180.6 MPa, 154.69 MPa, and 135.17 MPa for PFNA, “II” PFBN, and PFTBN, respectively. D-F represent the VMS distribution of the proximal femur after fixation with the corresponding devices, with peak values of 286.351 MPa, 257.79 MPa, and 229.84 MPa; PFTBN shows the most significant reduction in peak stress and stress distribution range.

The peak Von Mises Stress (VMS) of the PFNA with a 9 mm main nail was 180.6 MPa, that of the second-generation PFBN (II-PFBN) was 154.69 MPa, and that of the PFTBN was 135.17 MPa. Among the three devices, the PFNA exhibited the highest peak stress, while the PFTBN showed a significantly lower peak stress than the II-PFBN. Stress concentration areas were consistently located at the junction of the tension screw and the lateral wall, as well as the junction of the compression screw and the tension screw, with a wide distribution range of peak stress (Figure 3; Table 2).

**TABLE 2:** Results of displacement and von mises stress for three types of intramedullary nail-fracture models.

Main nail	Main nail 9 mm group			Main nail 11 mm group		
Device type	PFNA	“II”PFBN	PFTBN	PFNA	“II”PFBN	PFTBN
Femur displacement unit: mm	6.956	6.3365	5.9388	7.0895	6.6295	6.1585
Intramedullary nail displacement unit: mm	6.4735	6.0759	5.6587	6.8569	6.3477	5.9483
Femur stress unit: MPa	286.351	257.79	229.84	241.94	210.54	193.87
Intramedullary nail stress unit: MPa	180.6	154.69	135.17	257.68	225.82	204.49

#### 3.1.2 Von Mises Stress distribution of the femur

When fixed with the 9 mm main nail PFNA, the peak VMS of the proximal femur was 286.351 MPa; with the II-PFBN, it was 257.79 MPa; and with the PFTBN, it was 229.84 MPa. Compared with the PFNA, the II-PFBN reduced the peak femoral stress and narrowed the distribution range of peak stress at the femoral neck. The PFTBN showed a more significant reduction in the peak stress of the proximal femur and medial wall compared with the other two devices, while also narrowing their stress distribution ranges (Figure 3; Table 2).

#### 3.1.3 Displacement distribution of the three intramedullary nail devices

**FIGURE 4:**
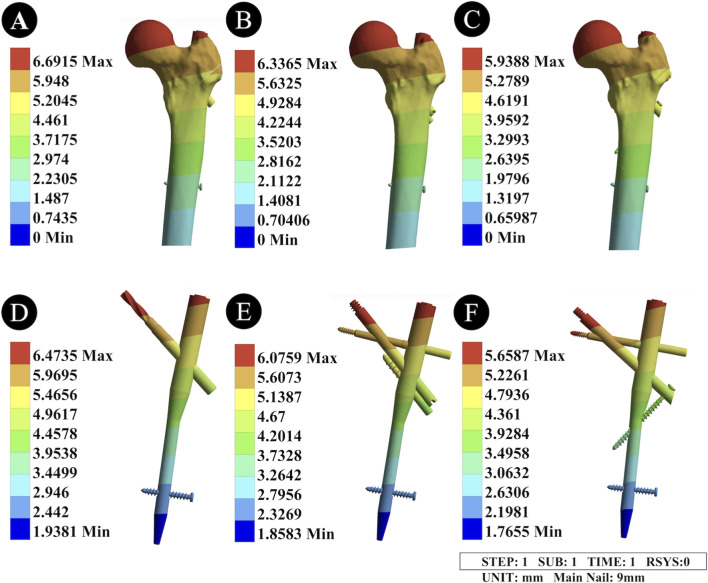
Displacement distribution diagrams of the femur and intramedullary nail devices under the unreamed condition. **(A,D)** Displacement distribution diagrams of the femur and fracture surfaces fixed with the PFNA device; **(B,E)** Displacement distribution diagrams of the femur and fracture surfaces fixed with the “II” PFBN device; **(C,F)** Displacement distribution diagrams of the femur and fracture surfaces fixed with the PFTBN device. **(A-C)** represent the displacement distribution of the three intramedullary nail devices, all concentrated at the top of the cephalomedullary nail; their peak displacements are 6.4735 mm (PFNA), 6.0759 mm (II-PFBN), and 5.6587 mm (PFTBN), respectively, with PFTBN showing the smallest peak displacement but a wider distribution range than II-PFBN. **(D-F)** represent the displacement distribution of the proximal femur after fixation with the corresponding devices, with peak displacements of 6.6915 mm (PFNA), 6.3365 mm (II-PFBN), and 5.9388 mm (PFTBN); both II-PFBN and PFTBN can enhance internal fixation stability and reduce femoral displacement.

The peak displacement of the 9 mm main nail PFNA was 6.4735 mm, that of the II-PFBN was 6.0759 mm, and that of the PFTBN was 5.6587 mm. The displacement distribution areas of the three intramedullary nails were consistent, all concentrated at the top of the cephalomedullary nail. The PFTBN had the smallest peak displacement but a wider displacement range than the II-PFBN (Figure 4; Table 2).

#### 3.1.4 Displacement distribution of the proximal and distal femur

When fixed with the 9 mm main nail PFNA, the peak displacement of the proximal femur was 6.956 mm; with the II-PFBN, it was 6.3365 mm; and with the PFTBN, it was 5.9388 mm. Both the II-PFBN and PFTBN could provide a more stable internal fixation structure, offering stronger support to the entire femur and reducing the degree of femoral displacement (Figure 4; Table 2).

### 3.2 Von Mises Stress and displacement distributions of three intramedullary nail devices and the femur under reamed condition

#### 3.2.1 Von Mises Stress distribution of the three intramedullary nail Devices

**FIGURE 5:**
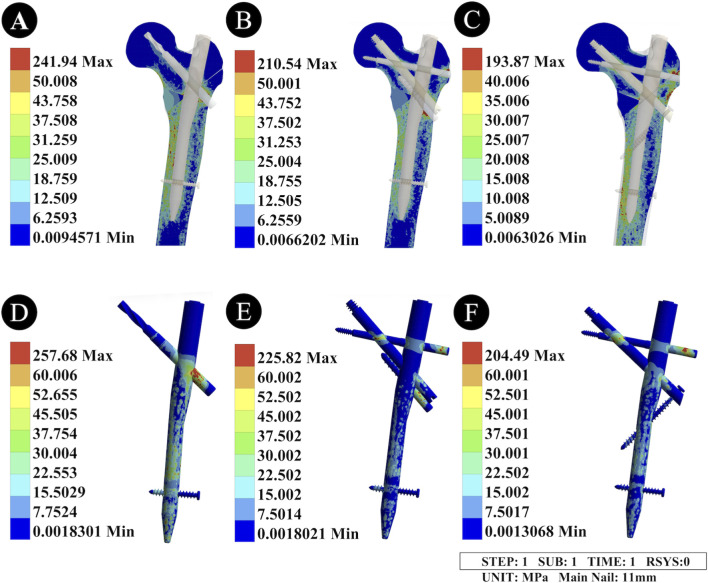
Von Mises Stress distribution diagrams of the femur and intramedullary nail devices under the reamed condition. **(A,D)** Stress distribution diagrams of the PFNA device; **(B,E)** Stress distribution diagrams of the “II” PFBN device; **(C,F)** Stress distribution diagrams of the PFTBN device. **(A-C)** represent the Von Mises stress (VMS) distribution of the three intramedullary nail devices (11 mm main nail), with stress concentration areas consistent with those under the unreamed condition; their peak VMS values are 257.68 MPa (PFNA), 225.82 MPa (II-PFBN), and 204.49 MPa (PFTBN), respectively, showing a significant increase compared with the unreamed condition. **(D-F)** represent the VMS distribution of the proximal femur after fixation with the corresponding devices, with peak values of 241.94 MPa (PFNA), 210.54 MPa (II-PFBN), and 193.87 MPa (PFTBN); reaming reduces the overall femoral peak stress, and PFTBN exhibits the most significant reduction in peak stress and stress distribution range of the proximal femur and medial wall.

The peak VMS of the PFNA with an 11 mm main nail was 257.68 MPa, that of the II-PFBN was 225.82 MPa, and that of the PFTBN was 204.49 MPa. Compared with the unreamed condition (9 mm main nail), reaming resulted in a significant increase in the peak stress of all three devices, and the stress concentration areas were consistent with those under the unreamed condition (Figure 5). Among the three devices, the PFNA still had the highest peak stress, while the PFTBN had a lower peak stress than the II-PFBN (Table 2).

#### 3.2.2 Von Mises Stress distribution of the femur

When fixed with the 11 mm main nail PFNA, the peak VMS of the proximal femur was 241.94 MPa; with the II-PFBN, it was 210.54 MPa; and with the PFTBN, it was 193.87 MPa. Compared with the unreamed condition, reaming led to an overall reduction in the peak femoral stress. The II-PFBN further narrowed the distribution range of stress at the femoral neck compared with the PFNA, and the PFTBN remained the device that most significantly reduced the peak stress and stress distribution range of the proximal femur and medial wall (Figure 5; Table 2).​

#### 3.2.3 Displacement distribution of the three intramedullary nail devices

**FIGURE 6:**
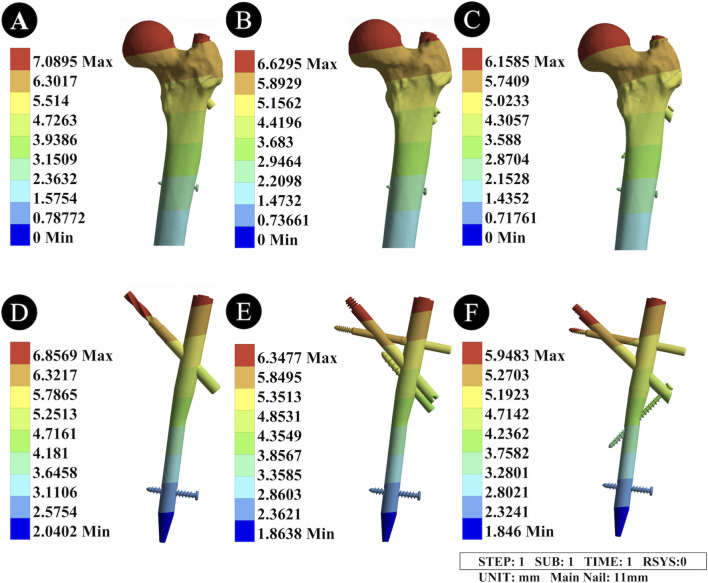
Displacement distribution diagrams of the femur and intramedullary nail devices under the reamed condition. **(A,D)** Displacement distribution diagrams of the femur and fracture surfaces fixed with the PFNA device; **(B,E)** Displacement distribution diagrams of the femur and fracture surfaces fixed with the “II” PFBN device; **(C,F)** Displacement distribution diagrams of the femur and fracture surfaces fixed with the PFTBN device. **(A-C)** represent the displacement distribution of the three intramedullary nail devices, all concentrated at the top of the cephalomedullary nail; their peak displacements are 6.8569 mm (PFNA), 6.3477 mm (II-PFBN), and 5.9483 mm (PFTBN), respectively, with a slight increase compared with the unreamed condition. PFTBN has the smallest peak displacement but a wider distribution range than II-PFBN. **(D-F)** represent the displacement distribution of the proximal femur after fixation with the corresponding devices, with peak displacements of 7.0895 mm (PFNA), 6.6295 mm (II-PFBN), and 6.1585 mm (PFTBN); reaming slightly increases femoral displacement, but both II-PFBN and PFTBN maintain good internal fixation stability and can effectively reduce femoral displacement.

The peak displacement of the 11 mm main nail PFNA was 6.8569 mm, that of the II-PFBN was 6.3477 mm, and that of the PFTBN was 5.9483 mm. Compared with the unreamed condition, reaming caused a slight increase in the peak displacement of all three devices, and the displacement distribution area remained concentrated at the top of the cephalomedullary nail. The PFTBN still had the smallest peak displacement but a wider displacement range than the II-PFBN (Figure 6; Table 2).

#### 3.2.4 Displacement distribution of the femur

When fixed with the 11 mm main nail PFNA, the peak displacement of the proximal femur was 7.0895 mm; with the II-PFBN, it was 6.6295 mm; and with the PFTBN, it was 6.1585 mm. Compared with the unreamed condition, reaming slightly increased the degree of femoral displacement. However, both the II-PFBN and PFTBN still maintained good internal fixation stability, which could effectively reduce femoral displacement (Figure 6; Table 2).

## 4 Discussion

This study thoroughly explored the therapeutic strategies for intertrochanteric femoral fractures complicated by lateral wall injury, with a specific focus on the application of intramedullary nail devices under reamed and unreamed conditions. When an intertrochanteric femoral fracture is combined with lateral wall injury, the supporting bone of the lateral femoral wall loses integrity, making intramedullary nail fixation prone to failure. Reconstructing the lateral wall has long been a key challenge in clinical treatment. Due to its advantages of minimal soft tissue damage, ease of operation, and stable nail fixation, intramedullary nailing has become the mainstream approach for treating intertrochanteric femoral fractures.

Among existing intramedullary nail devices, the PFNA is widely recognized by scholars as the gold standard for intertrochanteric femoral fracture treatment due to its unique merits. However, with the increasing incidence of high-energy fractures, the treatment of intertrochanteric femoral fractures has become increasingly complex. Previous studies have shown that PFNA is associated with a relatively high incidence of surgical complications, including nail migration, nail breakage, and femoral neck varus collapse (Lewis et al., 2022). These complications undoubtedly increase the risk of reoperation and mortality in elderly patients. The present study further revealed that PFNA exhibits stress concentration at the junction of the cephalomedullary nail and the main nail, which may be a critical factor contributing to clinical complications such as screw breakage and loosening. Additionally, this device is insufficient in supporting the lateral and medial femoral walls, failing to provide adequate biomechanical stability for intertrochanteric femoral fractures. Therefore, the development of intramedullary nail devices with superior biomechanical performance has become a current research focus.

With the advancement of biomechanical research, our understanding of different trabecular bone types and their mechanical distributions has become more comprehensive. The proximal femoral trabeculae include compressive trabeculae, tensile trabeculae, and intertrochanteric trabeculae, which collectively form Ward’s triangle to adapt to mechanical loads during various body postures and movements. Based on this understanding, Zhang et al. proposed the “I” PFBN (Zhang et al., 2025; Zhao et al., 2023; Fu et al., 2024). Modified from PFNA, this device incorporates a compression screw and a tension screw to form a stable triangular structure, thereby enhancing support for the medial and lateral femoral walls. Building on the successful clinical application of “I” PFBN, Zhang et al. further developed the “II” PFBN. The core design of “II” PFBN involves advancing the intersection of the lag screw and transverse screw, which significantly improves the fixation efficacy of the internal implant while reducing the stress shielding effect (Ding et al., 2023). Furthermore, the circular proximal structure of the main nail was modified to a flat-head design, effectively reducing the proximal volume of the main nail and minimizing the space-occupying interference on the proximal femoral cancellous bone after implantation. However, “II” PFBN has also demonstrated certain limitations in clinical applications and finite element analyses, particularly insufficient support for the lateral femoral wall, which may increase the risk of proximal femoral collapse deformity (Ding et al., 2023; Duan et al., 2024).

To overcome this limitation, Zhang et al. developed the PFTBN (Chen et al., 2024). By inserting an anchoring screw below the lesser trochanter at the tail of the compression screw of the “I” PFBN device, PFTBN overcomes the disadvantage of relying on fractured proximal femoral fragments of the lateral wall to resist the varus tendency of the cephalic cervical screw. Instead, the anchoring between the screw and the femoral shaft replaces the counterweight effect of the original lateral wall in resisting varus stress, reconstructing the lever resistance side of the lateral wall. Finite element analysis results showed that the PFTBN device can effectively disperse vertical loads, reduce the incidence of varus deformity, and improve overall stability by providing lateral wall support. Although the peak stress of the PFTBN device is relatively higher, its stress distribution is more reasonable, which helps reduce complications such as nail migration, breakage, and loosening.

PFNA exhibits stress concentration at the junction of the spiral blade and the main nail, a single force transmission node that is prone to local load accumulation. In contrast, the stress concentration of “II” PFBN and PFTBN is dispersed at the intersection of the compression screw and the main nail, as well as the junction of the tension screw tail and the lateral femoral wall. The multi-node stress distribution pattern effectively disperses local loads, reduces stress concentration, and thereby lowers the overall deformation and stress levels of the implant and femur. Among them, PFTBN demonstrated the optimal mechanical performance, suggesting that the layout design of its compression screw and tension screw is more consistent with the mechanical transmission needs after femoral fracture, enabling more reasonable load distribution and enhancing the stability of the internal fixation system.

Medullary canal reaming was first proposed by Kuntscher to increase the contact area between the intramedullary nail and bone, thereby improving stability (Bekos et al., 2021). Tucker et al. (2019) found that fracture type is one of the key factors affecting the fixation stability of proximal femoral fractures. The complexity of the fracture is negatively correlated with the movement tendency between fracture fragments; stable fracture types can reduce shear and axial movement at the fracture end. They also found that with the increase in intramedullary nail diameter, femoral stress concentration is effectively reduced, which is consistent with the results of this study.

The biomechanical analysis results of this study showed that increasing the intramedullary nail diameter presents a trend of reducing femoral stress while increasing internal fixation stress. This biomechanical characteristic suggests that a larger-diameter intramedullary nail may have greater supportive potential, facilitating stress transfer from the femur to the implant. Mechanically, this may help reduce the risk of femoral refracture caused by stress concentration, but this trend requires further verification with clinical data. The study also found that when using an 11 mm intramedullary nail for fixation, the maximum stress at the distal locking nail was lower than that with a 9 mm intramedullary nail across all fracture types. This result may be related to the larger nail-bone contact area provided by the larger-diameter intramedullary nail; the increase in this contact area can reduce the stress load on the distal locking nail from a biomechanical perspective, potentially laying a mechanical foundation for lowering the risk of fixation failure of the distal locking nail after internal fixation.

Significant differences in mechanical responses were observed for the same device with different nail diameters, showing a biomechanical trend that the deformation and stress levels corresponding to the 11 mm nail were higher than those of the 9 mm nail. The core reasons for this difference are associated with the contact area between the main nail and the medullary canal, as well as the mechanical transmission efficiency: the 11 mm nail has a larger diameter, increasing the contact area with the femoral medullary canal and enabling more direct load transmission. However, the increased rigidity of the implant also leads to more concentrated stress distribution at the contact interface. In contrast, the relatively larger gap between the 9 mm nail and the medullary canal can buffer local loads to a certain extent through slight displacement, reducing stress accumulation. Additionally, the increase in nail diameter may alter the force transmission path between the screws and the main nail, and this mechanical mechanism may further amplify the differences in mechanical performance among different devices.

All devices exhibited a consistent deformation distribution pattern: the maximum deformation at the femoral end was located at the head, gradually decreasing downward along the femur; at the intramedullary nail end, the maximum deformation occurred at the spiral blade, compression screw head, and tension screw head, gradually decreasing toward the distal end of the main nail. This distribution pattern is highly consistent with the mechanical load characteristics after femoral fracture—the femoral head, as the main stress-bearing part, bears the most concentrated axial and shear loads, while the screw heads, as the direct connection points between the implant and the femur, are key nodes for load transmission. This biomechanical distribution characteristic suggests that in clinical applications, attention may be paid to the fixation effect of the femoral head and screw heads, providing a reference direction from a mechanical perspective to avoid loss of fracture reduction or internal fixation loosening due to excessive local deformation.

From a biomechanical performance perspective, the PFTBN device exhibited the lowest deformation and stress levels under both nail diameters, with more dispersed stress distribution, showing superior mechanical characteristics related to internal fixation stability. This trend suggests that it may be more suitable for clinical scenarios involving severely comminuted fractures with high mechanical requirements for fixation stability. The mechanical performance of the second-generation PFBN (II-PFBN) was intermediate between that of the PFNA and PFTBN; its biomechanical characteristics demonstrate potential advantages in balancing stability and operational convenience, and it may serve as an alternative option for relevant clinical scenarios. In contrast, the mechanical performance of the PFNA was relatively weaker in the biomechanical analysis. The mechanical characteristics of its single spiral blade design suggest that this device may be more applicable to scenarios where the blood supply at the fracture end is poor, but the fracture reduction quality is good and the mechanical requirements for load transmission are low.

The selection of intramedullary nail diameter can refer to the biomechanical needs corresponding to the patient’s medullary canal morphology and fracture type: for patients with a wide medullary canal and good bone quality, the biomechanical characteristics of the 9 mm nail are more conducive to balancing the needs of fixation stability and stress buffering; for patients with a narrow medullary canal and unstable fracture ends, although the 11 mm nail has a higher stress level, its stronger supportive effect brought by the larger contact area may help reduce the risk of fracture displacement from a biomechanical perspective, providing mechanical reference for clinical selection.

This study compared the mechanical performance of three intramedullary nail devices for the fixation of intertrochanteric femoral fractures with lateral wall injury under both reamed and unreamed conditions via biomechanical analysis. The identified biomechanical trends of the results can provide valuable references for the selection of clinical surgical protocols. However, this study has certain limitations that may affect the interpretation of its findings: the research scope was limited to fractures with a 10 mm lateral wall thickness and two medullary canal diameters, restricting the generalizability of the results to all relevant fractures; the material parameters of the model were idealized and deviated from the actual biomechanical properties of human bone, which may lead to inconsistencies between the simulated data and clinical reality; physiological loads from hip muscle groups were not incorporated into the experimental loading conditions, resulting in a discrepancy between the simulated mechanical environment and the *in vivo* physiological state; additionally, finite element analysis is a deterministic simulation method that is not suitable for inferential statistics, making it impossible to verify the statistical significance and individual generalizability of the results, and thus the observed mechanical trends should not be regarded as definitive conclusions.

Future studies will further expand the research scope to include more fracture types (e.g., with different lateral wall thicknesses and comminution degrees) and intramedullary nail devices, optimize the material parameters of the model to better match the actual properties of human bone, improve the experimental loading conditions by incorporating the effects of muscle groups, and validate the findings with clinical data. This work aims to provide a more comprehensive and reliable biomechanical basis for clinical practice and the optimization of intramedullary nail device design.

## 5 Conclusion

This study demonstrated that the design of using a large-diameter intramedullary nail with reaming may reduce the mechanical risk of femoral refracture induced by stress concentration through the mechanical mechanism of increasing the nail-bone contact area. For intramedullary nail devices adapted to different medullary canal specifications for the treatment of intertrochanteric femoral fractures complicated with lateral wall injury, the structural form of PFNA presents more prominent biomechanical deficiencies from a design perspective. The structural design of the second-generation PFBN (II-PFBN) can narrow the stress concentration area between screws, which may reduce the mechanical predisposing factors for screw fracture and loosening. In comparison with the second-generation PFBN, the structural design of PFTBN enables a more uniform stress distribution among screws and exhibits superior performance in the mechanical design for lateral femoral wall support over the other two devices. This study provides a biomechanical reference direction for the subsequent structural design and optimization of intramedullary nail devices, and offers a mechanical basis for the improvement of nail diameter selection, screw layout and lateral wall support structure in device design.

## Data Availability

The original contributions presented in the study are included in the article/supplementary material, further inquiries can be directed to the corresponding authors.

## References

[B1] Baca V. Horak Z. Mikulenka P. Dzupa V. (2008). Comparison of an inhomogeneous orthotropic and isotropic material models used for FE analyses. Med. Eng. Phys. 30 (7), 924–930. 10.1016/j.medengphy.2007.12.009 18243761

[B2] Bekos A. Sioutis S. Kostroglou A. Saranteas T. Mavrogenis A. F. (2021). The history of intramedullary nailing. Int. Orthop. 45 (5), 1355–1361. 10.1007/s00264-021-04973-y 33575858

[B3] Brox W. T. Roberts K. C. Taksali S. Wright D. G. Wixted J. J. Tubb C. C. (2015). The American academy of orthopaedic Surgeons Evidence-Based Guideline on management of hip fractures in the elderly. J. Bone Jt. Surg. Am. 97 (14), 1196–1199. 10.2106/JBJS.O.00229 26178894 PMC6948785

[B4] Chen P. Fan Z. Xu N. Wang H. (2023). A biomechanical investigation of a novel intramedullary nail used to salvage failed internal fixations in intertrochanteric fractures. J. Orthop. Surg. Res. 18 (1), 632. 10.1186/s13018-023-04112-w 37641046 PMC10463605

[B5] Chen X. Tang M. Zhang X. Zhang Y. Wang Y. Xiong C. (2024). A novel internal fixation design for the treatment of AO/OTA-31A3.3 intertrochanteric fractures: finite element analysis. Orthop. Surg. 16 (7), 1684–1694. 10.1111/os.14041 38784971 PMC11216835

[B6] Ding K. Zhu Y. Zhang Y. Li Y. Wang H. Li J. (2023). Proximal femoral bionic nail-a novel internal fixation system for the treatment of femoral neck fractures: a finite element analysis. Front. Bioeng. Biotechnol. 11, 1297507. 10.3389/fbioe.2023.1297507 38116197 PMC10728673

[B7] Duan W. Liang H. Fan X. Zhou D. Wang Y. Zhang H. (2024). Research progress on the treatment of Geriatric intertrochanteric femur fractures with proximal femur bionic nails (PFBNs). Orthop. Surg. 16 (10), 2303–2310. 10.1111/os.14134 38982572 PMC11456711

[B8] Fan J. Xu X. Zhou F. Zhang Z. Tian Y. Ji H. (2021). Risk factors for implant failure of intertrochanteric fractures with lateral femoral wall fracture after intramedullary nail fixation. Injury 52 (11), 3397–3403. 10.1016/j.injury.2021.07.025 34321191

[B9] Fan J. Xu X. Zhou F. (2022). The lateral femoral wall thickness on the risk of post-operative lateral wall fracture in intertrochanteric fracture after DHS fixation: a finite element analysis. Injury 53 (2), 346–352. 10.1016/j.injury.2021.11.015 34789386

[B10] Fu H. Hu L. Zou F. Liao X. Zheng Y. Jin P. (2024). A comparative Study of the early postoperative outcome of three intramedullary fixation modalities in the treatment of intertrochanteric fractures of the femur in the elderly. J. Musculoskelet. Neuronal Interact. 24 (3), 310–317. 39219329 PMC11367175

[B11] Grønhaug K. M. L. Dybvik E. Matre K. Östman B. Gjertsen J. E. (2022). Intramedullary nail *versus* sliding hip screw for stable and unstable trochanteric and subtrochanteric fractures: 17,341 patients from the Norwegian Hip Fracture Register. Bone Jt. J. 104-B (2), 274–282. 10.1302/0301-620X.104B2.BJJ-2021-1078.R1 35094569

[B12] Haidukewych G. J. (2009). Intertrochanteric fractures: ten tips to improve results. J. Bone Jt. Surg. Am. 91 (3), 712–719. 19255235

[B13] Hsu C. E. Shih C. M. Wang C. C. Huang K. C. (2013). Lateral femoral wall thickness. A reliable predictor of post-operative lateral wall fracture in intertrochanteric fractures. Bone Jt. J. 95-B (8), 1134–1138. 10.1302/0301-620X.95B8.31495 23908432

[B14] Huang Q. Xu Y. Xue H. Wang Q. Li M. Ren C. (2022). Percutaneous reduction with double screwdrivers *versus* limited open reduction in the treatment of irreducible extracapsular hip fractures. BMC Musculoskelet. Disord. 23 (1), 429. 10.1186/s12891-022-05390-x 35524242 PMC9077818

[B15] Im G. I. Shin Y. W. Song Y. J. (2005). Potentially unstable intertrochanteric fractures. J. Orthop. Trauma 19 (1), 5–9. 10.1097/00005131-200501000-00002 15668577

[B16] Kokoroghiannis C. Aktselis I. Deligeorgis A. Fragkomichalos E. Papadimas D. Pappadas I. (2012). Evolving concepts of stability and intramedullary fixation of intertrochanteric fractures--a review. Injury 43 (6), 686–693. 10.1016/j.injury.2011.05.031 21752370

[B17] Lewis S. R. Macey R. Lewis J. Stokes J. Gill J. R. Cook J. A. (2022). Surgical interventions for treating extracapsular hip fractures in older adults: a network meta-analysis. Cochrane Database Syst. Rev. 2 (2), CD013405. 10.1002/14651858.CD013405.pub2 35142366 PMC8830342

[B18] Li J. Tang S. Zhang H. Li Z. Deng W. Zhao C. (2019). Clustering of morphological fracture lines for identifying intertrochanteric fracture classification with Hausdorff distance-based K-means approach. Injury 50 (4), 939–949. 10.1016/j.injury.2019.03.032 31003702

[B19] Li S. Su Z. H. Zhu J. M. Sun W. J. Zhu Y. C. Wang J. (2023). The importance of the thickness of femoral lateral wall for treating intertrochanteric fractures: a finite elements analysis. Sci. Rep. 13 (1), 12679. 10.1038/s41598-023-39879-9 37542169 PMC10403567

[B20] Mohebbi S. Amiri A. Nabian M. H. (2025). Proximal femoral bionic nail (PFBN) offers comparable functional and clinical outcomes to PFNA and intertan in the treatment of intertrochanteric fractures: a systematic review and meta-analysis. J. Orthop. Surg. Res. 20 (1), 937. 10.1186/s13018-025-06395-7 41163169 PMC12574036

[B21] Pairon P. Ossendorf C. Kuhn S. Hofmann A. Rommens P. M. (2015). Intramedullary nailing after external fixation of the femur and tibia: a review of advantages and limits. Eur. J. Trauma Emerg. Surg. 41 (1), 25–38. 10.1007/s00068-014-0448-x 26038163

[B22] Palm H. Jacobsen S. Sonne-Holm S. Gebuhr P. (2007). Integrity of the lateral femoral wall in intertrochanteric hip fractures: an important predictor of a reoperation. J. Bone Jt. Surg. Am. 89 (3), 470–475. 10.2106/JBJS.F.00679 17332094

[B23] Papini M. Zdero R. Schemitsch E. H. Zalzal P. (2007). The biomechanics of human femurs in axial and torsional loading: comparison of finite element analysis, human cadaveric femurs, and synthetic femurs. J. Biomech. Eng. 129 (1), 12–19. 10.1115/1.2401178 17227093

[B24] Penzkofer R. Maier M. Nolte A. von Oldenburg G. Püschel K. Bühren V. (2009). Influence of intramedullary nail diameter and locking mode on the stability of tibial shaft fracture fixation. Arch. Orthop. Trauma Surg. 129 (4), 525–531. 10.1007/s00402-008-0700-0 18654791

[B25] Queally J. M. Harris E. Handoll H. H. Parker M. J. (2014). Intramedullary nails for extracapsular hip fractures in adults. Cochrane Database Syst. Rev. 2014 (9), CD004961. 10.1002/14651858.CD004961.pub4 25212485 PMC10835205

[B26] Rosa N. Marta M. Vaz M. Tavares S. M. O. Simoes R. Magalhães F. D. (2019). Intramedullary nailing biomechanics: evolution and challenges. Proc. Inst. Mech. Eng. H. 233 (3), 295–308. 10.1177/0954411919827044 30887900

[B27] Tucker S. M. Wee H. Fox E. Reid J. S. Lewis G. S. (2019). Parametric finite element analysis of intramedullary nail fixation of proximal femur fractures. J. Orthop. Res. 37 (11), 235–236. 10.1002/jor.24401 31254411

[B28] Xu B. Y. Yan S. Low L. L. Vasanwala F. F. Low S. G. (2019). Predictors of poor functional outcomes and mortality in patients with hip fracture: a systematic review. BMC Musculoskelet. Disord. 20 (1), 568. 10.1186/s12891-019-2950-0 31775693 PMC6882152

[B29] Zhang Y. Li C. Shi X. Gao Q. (2025). The clinical efficacy of proximal femoral nail antirotation and proximal femoral bionic nail in the treatment of intertrochanteric fractures of the femur in the elderly: a systematic review and meta-analysis. Jt. Dis. Relat. Surg. 36 (3), 522–534. 10.52312/jdrs.2025.2302 40783984 PMC12456339

[B30] Zhao H. Deng X. Liu W. Chen W. Wang L. Zhang Y. (2023). Proximal femoral bionic nail (PFBN)-an innovative surgical method for unstable femoral intertrochanteric fractures. Int. Orthop. 47 (4), 1089–1099. 10.1007/s00264-023-05696-y 36719445

